# Role of affinity in plasma cell development in the germinal center light zone

**DOI:** 10.1084/jem.20231838

**Published:** 2023-11-08

**Authors:** Mohamed A. ElTanbouly, Victor Ramos, Andrew J. MacLean, Spencer T. Chen, Maximilian Loewe, Sandra Steinbach, Tarek Ben Tanfous, Brianna Johnson, Melissa Cipolla, Anna Gazumyan, Thiago Y. Oliveira, Michel C. Nussenzweig

**Affiliations:** 1 Laboratory of Molecular Immunology, The Rockefeller University, New York, NY, USA; 2 Immunology Program, Sloan Kettering Institute, Memorial Sloan Kettering Cancer Center, New York, NY, USA; 3 Howard Hughes Medical Institute, The Rockefeller University, New York, NY, USA

## Abstract

Protective immune responses to many pathogens depend on the development of high-affinity antibody-producing plasma cells (PC) in germinal centers (GCs). Transgenic models suggest that there is a stringent affinity-based barrier to PC development. Whether a similar high-affinity barrier regulates PC development under physiologic circumstances and the nature of the PC fate decision has not been defined precisely. Here, we use a fate-mapping approach to examine the relationship between GC B cells selected to undergo additional rounds of affinity maturation, GC pre-PC, and PC. The data show that initial PC selection overlaps with GC B cell selection, but that the PC compartment accumulates a less diverse and higher affinity collection of antibodies over time. Thus, whereas the GC continues to diversify over time, affinity-based pre-PC selection sieves the GC to enable the accumulation of a more restricted group of high-affinity antibody-secreting PC.

## Teaser

We show in polyclonal settings that GC B cell differentiation into plasma cells is affinity dependent, resulting in the generation of high-affinity serum responses. We profile GC cells poised for PC differentiation compared with GC B cells selected for further affinity maturation.

## Introduction

Increasing antibody affinity over time is a hallmark feature of humoral immune responses that is associated with increasing levels of neutralizing activity and is essential for optimal vaccine efficacy (Eisen and Siskind, 1964; Bannard and Cyster, 2017; Victora and Nussenzweig, 2022). High-affinity antibodies are typically acquired in germinal centers (GCs) (Victora and Nussenzweig, 2022; Inoue and Kurosaki, 2023), which are microanatomical features of B cell follicles in lymphoid organs. GCs are divided into two zones: a dark zone (DZ), wherein B cells undergo extensive cell division and somatic hypermutation of their antibody genes, and a light zone (LZ) where rare B cells expressing antibodies that carry high-affinity mutations are selected for additional rounds of clonal expansion and mutation in the DZ (Allen et al., 2004; Röhlich, 1930; Victora and Nussenzweig, 2022; Inoue and Kurosaki, 2023).

Somatic mutation is a random process that can both enhance or more frequently damage antibody genes. Thus, approximately half of the B cells participating in a GC reaction die every 6 h due to mutational damage or absence of positive selection (Inoue and Kurosaki, 2023; Liu et al., 1989; Mayer et al., 2017). The remainder will either be selected to differentiate into memory B cells, continue to cycle between LZ and DZ, or become plasma cells (PC) (Inoue and Kurosaki, 2023; Nutt et al., 2015; Suan et al., 2017b; Victora and Nussenzweig, 2022). GC B cells destined for the memory B cell compartment express a distinct set of transcription factors including *Bach2*, *Hhex*, and *Tle3* (Laidlaw et al., 2020; Shinnakasu et al., 2016), and display a lower affinity for antigen than LZ B cells selected for DZ re-entry and continued expansion and mutation (Shinnakasu et al., 2016; Smith et al., 2000; Suan et al., 2017a; Viant et al., 2020; Wong et al., 2020).

Selection for DZ re-entry is mediated by a combination of T cell help and B cell receptor (BCR) signaling (Chen et al., 2023; Victora and Nussenzweig, 2022; Victora et al., 2010). LZ B cells expressing antibodies with relatively higher affinity capture and endocytose more cognate antigen than their counterparts, and process and present it to a limiting number of T follicular helper cells (Ise et al., 2018; Victora et al., 2010). Concurrent BCR-mediated signals prolong B cell LZ residence and increase the probability of engaging helper T cells (Chen et al., 2023; Ise et al., 2018). The PC program is thought to be induced in GC B cells that develop relatively higher affinity antibodies (Kräutler et al., 2017; Phan et al., 2006; Radbruch et al., 2006; Shih et al., 2002a). The idea that PC development is restricted to GC B cells with the highest affinity is supported by experiments in mice that carry the anti-hen egg lysozyme transgene (Kräutler et al., 2017; Phan et al., 2006). In these mice, acquisition of a point mutation that increases affinity by ∼100-fold induces LZ B cells to express *Irf4* initiating PC development (Phan et al., 2006) and a transcriptional program that ultimately supports a secretory compartment capable of producing up to 10^4^ antibody molecules per second (Hibi and Dosch, 1986; Nutt et al., 2015; Radbruch et al., 2006). But whether and how selection for PC development or GC re-entry differ, and how affinity impacts PC development in a polyclonal setting are incompletely understood.

Here, we used an unbiased fate-mapping approach to examine how differences in affinity mediate selection for continued GC residence and PC development in mice with a polyclonal immune system immunized with a model antigen 4-hydroxy-3-nitrophenyl acetyl conjugated to ovalbumin (NP-Ova).

## Results

### Lineage tracing GC B and PC

To examine GC development, we permanently labeled activated and GC B cells using a combination of tamoxifen-inducible *S1pr2*-CreERT2 and Rosa26-lox-stop-lox-TdTomato (S1pr2.Cre^ERT2^.TdT) (Shinnakasu et al., 2016). In this system, PC that subsequently develop either from activated or GC B cells will also be permanently labeled.

**Figure 1.**
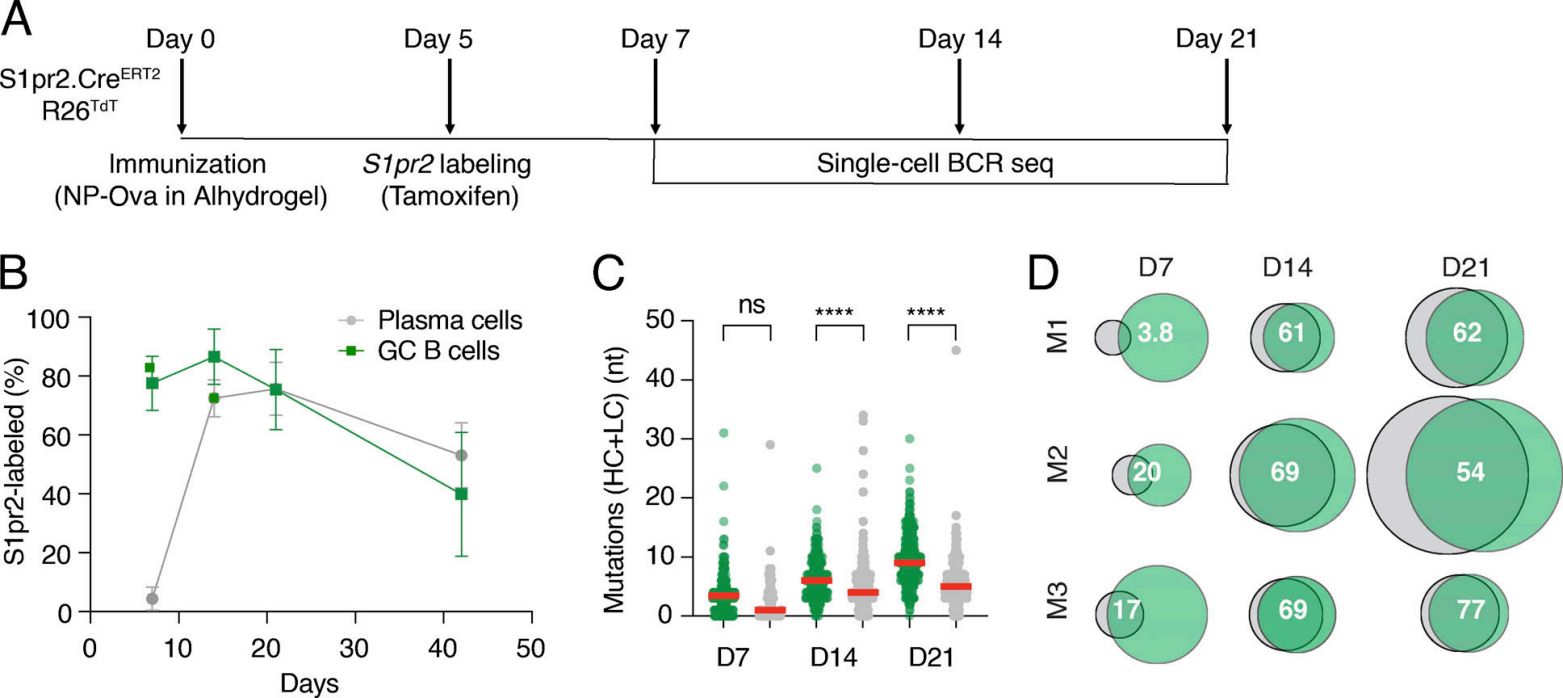
**NP-Ova immunization. (A)** Schematic representation of the experiment: S1pr2CreERT2.R26TdTomato mice were immunized with NP-Ova in alhydrogel on day 0 followed by tamoxifen administration via oral gavage on day 5. FACS sorting and analysis were performed on days 7, 14, and 21. **(B)** Graph shows the kinetics of *S1pr2* labeling of GC B cells (green) and their PC progeny (gray). Error bars denote the standard deviation of mean values (dots). Kruskal–Wallis test with Dunn’s multiple comparisons analysis was performed for the different timepoints for each compartment. Data represents three experiments with three to four mice each. Significant differences in *S1pr2*-labeled cells exist in GC B cells between days 21 and 42 (P value, 0.0049) and PCs between days 7 and 14 (P value, 0.0288). **(C)** Graph shows the median number of mutations (SHM) for both the heavy and light chains (HC + LC) at each time point. The red bar denotes the median value and the statistical significance of * denotes P ≤ 0.05, **P ≤ 0.01, ***P ≤ 0.001, and ****P ≤ 0.0001, whereas “ns” indicates no significant differences. **(D)** Euler plots showing calculated percentage of clonal overlap between the GC B cells (green) and PCs (gray) for each representative mouse for the three time points analyzed. The size of the plot is proportional to the total number of sequenced cells per compartment and the size of the overlap region is proportional to the number of shared clones. All sequences including unique sequences were included and clones with related sequences were counted once irrespective of size. The numbers indicate the percentage clonal overlap. Data represent three experiments with three to four mice each.

**Figure S1.**
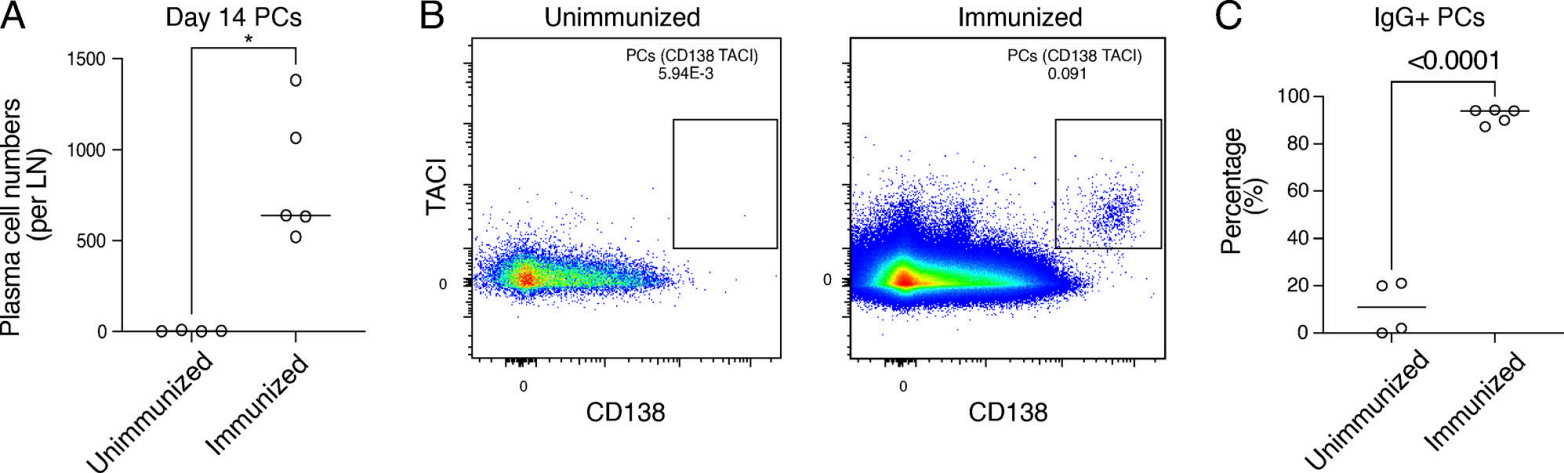
**Popliteal lymph nodes are devoid of PC in unimmunized mice.** Related to Fig. 1. **(A)** Number of PC in the popliteal LNs of unimmunized mice or immunized mice on day 14 after immunization which defines the peak of the GC response. A statistical significance of * denotes P ≤ 0.05. **(B)** Representative flow plots of data quantified in A. **(C)** Summary of percentage IgM^+^ or IgG^+^ PCs on day 14 among the S1pr2-labeled cells.

To validate this approach, we immunized mice with NP-Ova and administered a single dose of tamoxifen by gavage on day 5 after immunization (Fig. 1 A). Popliteal lymph node (LN) GC and PC were examined on days 7, 14, 21, and 42 after immunization (Madisen et al., 2010). In the absence of immunization, there were no detectable GCs or PC in the popliteal LN (Viant et al., 2021b) (Fig. S1, A and B). In contrast, GCs were readily detected in draining LNs after immunization, ∼80% and 90% of which were labeled on days 7 and 14, respectively (Fig. 1 B). There was a significant decline in labeled cells by day 42, which is likely to be due to late GC invasion by unlabeled naïve B cells (de Carvalho et al., 2023; Hägglöf et al., 2023; Schwickert et al., 2007; Fig. 1 B). As might be expected, labeling was nearly absent from the PC compartment on day 7 but reached a peak of nearly 75% on day 21 with the majority expressing IgG, suggesting a GC origin (Fig. 1 B and Fig. S1 C). The difference in labeling kinetics between the GC and PC compartments is likely a reflection of the time it takes for PC to develop from GC cells.

**Figure 2.**
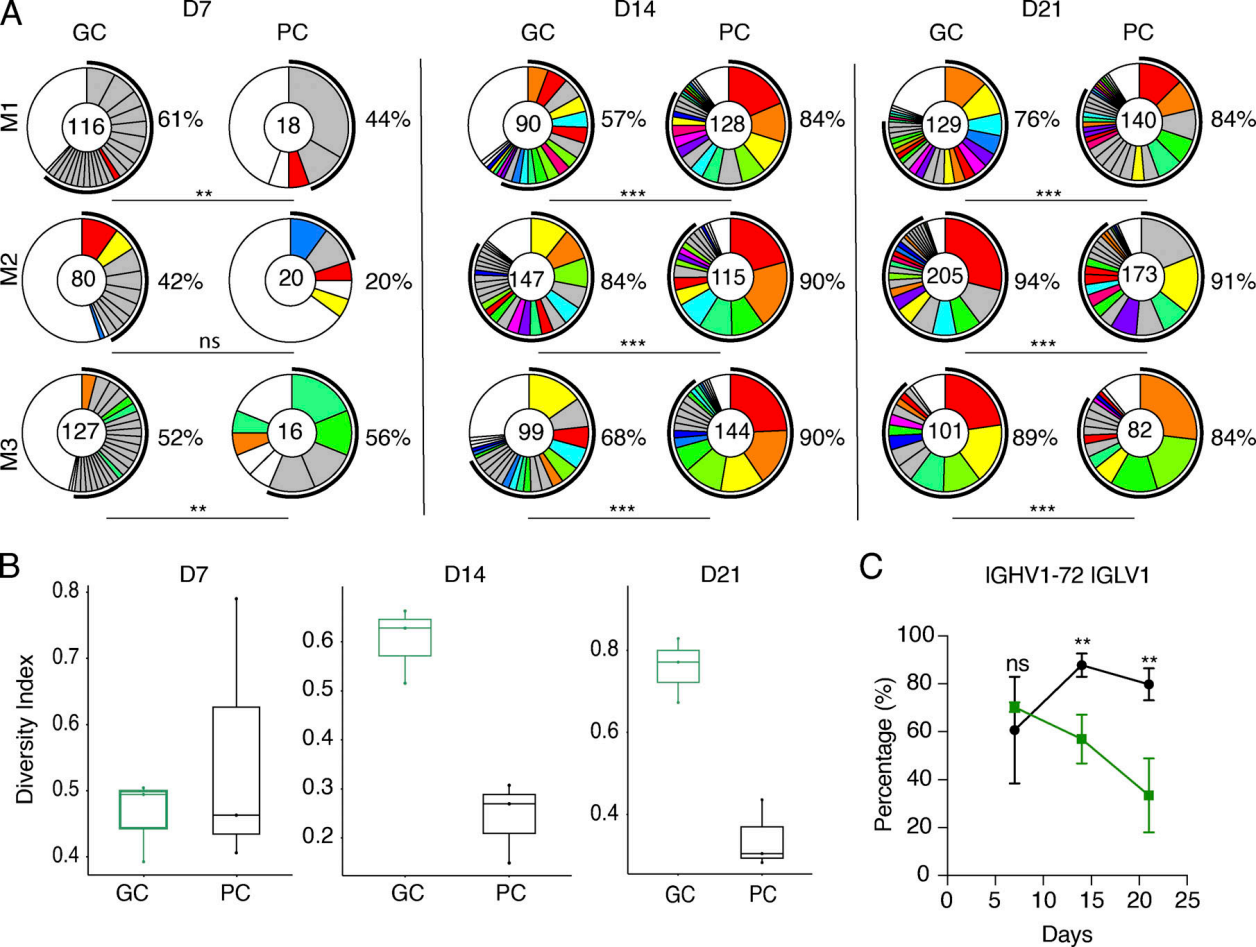
**Antibody clonal analysis. (A)** Clonal overlap between contemporaneous GC B cells and PC: Pie charts showing the distribution of antibody sequences obtained for GC B and PC from individual LNs from three mice on days 7, 14, and 21 after immunization. The number inside the circle indicates the number of sequences analyzed. Slice size is proportional to the number of clonally related sequences, with the fraction of clonally expanded sequences summarized as percentage (black outline and associated numbers). Colored slices indicate shared clones between GC B and PC (same IGHV and IGLV genes, with highly similar complementarity-determining region 3 sequences [CDR3s]), gray slices indicate clones unique to the time point and white slices indicate sequences isolated only once per time point. **(B)** Graphs show Simpson's diversity index (SDI) analysis (Simpson, 1949) for all the antibody sequences shown in A on days 7, 14, and 21 after immunization. **(C)** Timeline graph quantifies the percentage of cells carrying the IGHV1-72*01 heavy chain paired with IGLV1 within the labeled GC B cell or PC compartment. Statistical significance of ** indicates P ≤ 0.01 and *** indicates P ≤ 0.001. Data represents three experiments with three to four mice each.

To determine the relationship between the GC B cells and developing PC, we purified single *S1pr2*-labeled cells from individual LNs on days 7, 14, and 21 after immunization and sequenced their antibody genes (Fig. 1 A). Consistent with the delay in labeled PC emergence from GCs, the number of somatic mutations in PC was significantly lower than in GC B cells obtained at the same time point (Fig. 1 C). Despite this difference, when all sequences were considered, there was extensive clonal overlap ranging from 61 to 69% and 60 to 75% on days 14 and 21 after immunization (Fig. 1 D). However, when the relative size of the expanded clones and all non-clonal single sequences in the two compartments were considered, we found significant differences that were especially evident at the two later time points (Fig. 2 A). Simpson's diversity index analysis revealed significantly higher levels of diversity in the GC compartment relative to PC on days 14 and 21 after immunization (Fig. 2 B). The increased diversity in GC B cells is in part the result of activation-induced cytidine deaminase (AID) expression. The absence of AID in PCs that are continuing to undergo cell division is likely to explain the difference in the diversity index between the two cell types.

Further Ig sequence analysis revealed that on day 7 after immunization 60–70% of all *S1pr2*-labeled GC B and PC expressed the IGHV1-72*01 Ig heavy chain that is associated with high-affinity NP-binding activity (Allen et al., 1988; Fig. 2 C). Consistent with increasing GC diversity over time, the relative frequency of IGHV1-72*01 usage declined among *S1pr2*-labeled GC B cells after day 14 (de Carvalho et al., 2023; Hägglöf et al., 2023; Smith et al., 1985). In contrast, relatively high levels of IGHV1-72*01 usage persisted in the PC compartment (Fig. 2 C). The difference in IGHV1-72*01 usage between the two compartments suggests a process that favors diversification in the GC B cell repertoire and persistent selection for affinity among PC. In conclusion, B cells selected to become PC are generally representative of the GC but less diverse.

### GC B and PC antibody affinity

**Figure 3.**
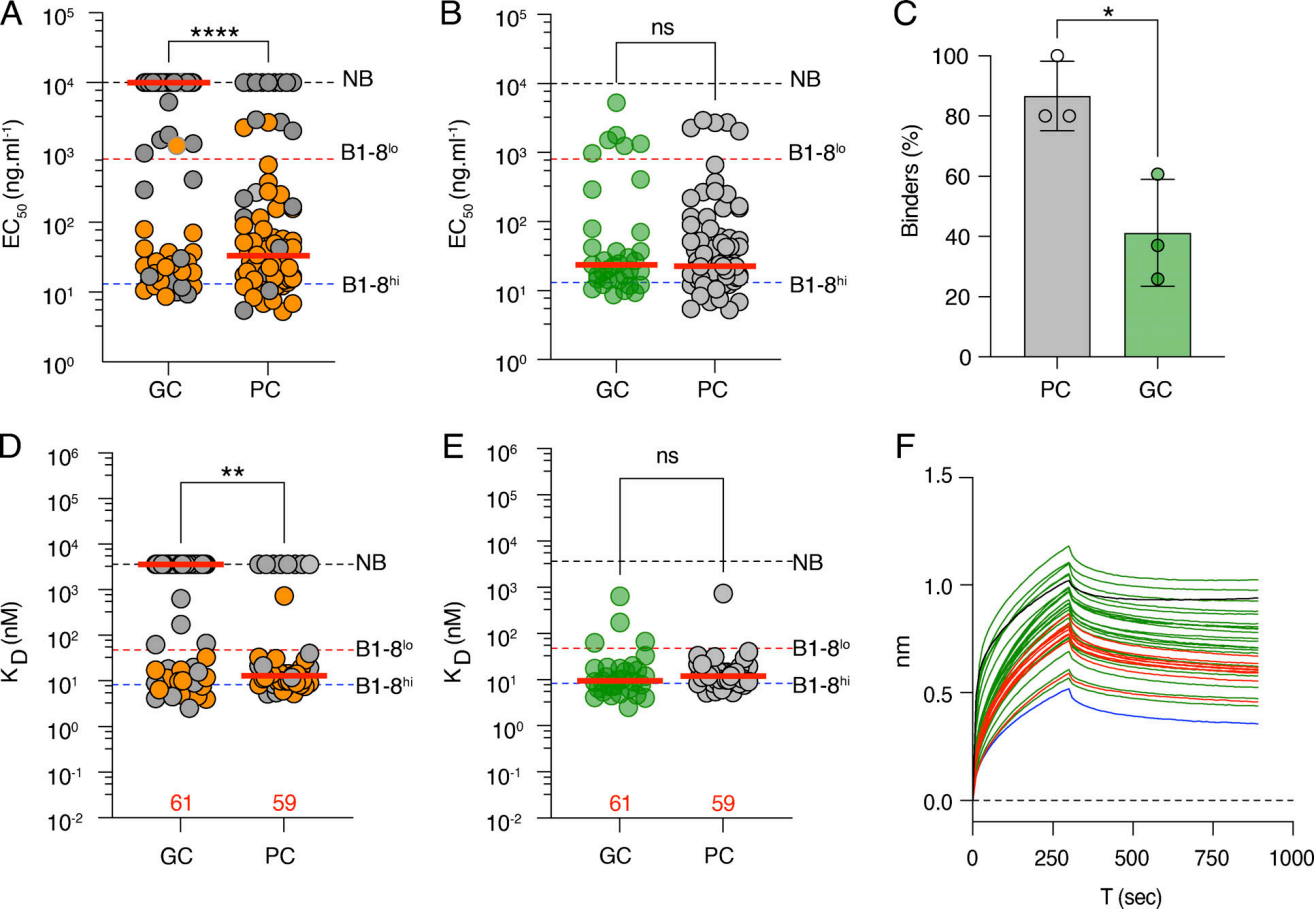
**Antibody affinity.** **(A)** Dot plot showing ELISA EC_50_ binding for 81 and 86 representative Fabs expressed and tested from GC B and PC, respectively, on day 21 after immunization. VH1-72*01 antibodies are in orange, and all other antibodies are in gray. **(B)** As in A, but excluding antibodies that do not bind. GC B (green); PC (gray). **(C)** Bar graph shows the fraction of all Fabs tested that show demonstrable binding in ELISA. Statistical significance was determined using an unpaired *t* test. **(D)** Graph shows monovalent binding affinity K_D_ (nM) determined by BLI for 61 and 59 representative Fabs obtained from GC B and PC, respectively, on day 21 after immunization. Color scheme as in A. **(E)** As in D, but excluding Fabs that do not bind. The red horizontal bars in A–E represent the median values. Statistical significance was determined using two-tailed Mann–Whitney U-tests comparing the differences between GC and PC EC_50_ in A and B, and K_D_ in D and E. **(F)** Graphs show BLI traces for 43 GC B (green) and PC (red) Fabs that show no demonstrable binding activity under monovalent conditions (D) tested under multivalent conditions. B1-8lo (black) and negative control Fab (blue). All experiments in this figure were performed at least in duplicate. The red bar denotes the median value and the statistical significance of * denotes P ≤ 0.05, **P ≤ 0.01, ***P ≤ 0.001, and ****P ≤ 0.0001, whereas “ns” indicates no significant differences.

To compare the affinity of the BCRs expressed by developing GC B and PC, we produced 166 representative antigen-binding fragments (Fabs) from fate-mapped cells from the day 21 time point (Fig. 1 A and Table S1). The relative binding activity of each antibody was initially assessed by measuring the half-maximal effective concentrations (EC_50_) by enzyme-linked immunosorbent assay (ELISA) against the NP-Ova immunogen (Fig. 3 A). We found that antibodies cloned from GC B cells had a significantly higher median EC_50_ than their PC counterparts suggesting lower affinities (10,000 ng/ml versus 33 ng/ml, respectively; Fig. 3 A). The difference was primarily due to the greater number of non-binders found in the GC B than the PC compartment (40% versus 85%; Fig. 3, A–C). As might be expected, the difference in ELISA-binding activity was directly associated with the relative enrichment of IGHV1-72*01 usage in the PC compartment (Fig. 2 C and Fig. 3 A). However, when the analysis was restricted to antibodies with measurable binding activity the two compartments were similar (Fig. 3 B).

To obtain quantitative information on antibody affinity, we performed bio-layer interferometry (BLI) experiments wherein NP-Ova was immobilized on the biosensor chip and exposed to the Fab in solution (Fig. 3, D and E). B1-8^hi^ and B1-8^lo^ and Neg control Fabs were used as high- and low-affinity and negative control references (Chen et al., 2023; Shih et al., 2002b). The BLI affinity measurements confirmed the ELISAs and showed K_D_ (equilibrium dissociation constant) measurements ranging from non-measurable to 4 nM (Fig. 3, D and E). Notably ∼50% and 15% of the GC B and PC Fabs failed to show measurable binding under monomeric binding conditions, respectively (Fig. 3, D and E).

The interaction between the BCR and antigen displayed as immune complexes on follicular dendritic cells in GCs is likely to be multimeric. To model higher valency interactions, we performed BLI experiments wherein Fabs that failed to show binding under monomeric conditions were immobilized on the biosensor chip and exposed to soluble NP_16_-Ova (Fig. 3 F). Of the 43 Fabs that failed to bind under monomeric conditions, 39 demonstrated binding under multivalent conditions (Fig. 3 F). Thus, nearly all the antibodies expressed by GC B cells bind to the immunogen, but a significant fraction do so with relatively low apparent affinities that are only measurable under multivalent conditions. Many of the cells expressing these antibodies cannot be captured by traditional antigen-baiting methods using tetramers (Chen et al., 2023; Viant et al., 2020). We conclude that in mice with an intact immune system responding to immunization with NP-Ova, nearly 50% of all GC B cells found in draining LNs bind to antigen with lower affinities than PC. Conversely, when the analysis is restricted to higher affinity antibodies that bind under monovalent conditions, 88% of the antibodies produced by PC show affinities that are indistinguishable from those produced by their GC B cell counterparts.

### Contemporaneous GC B and PC

**Figure 4.**
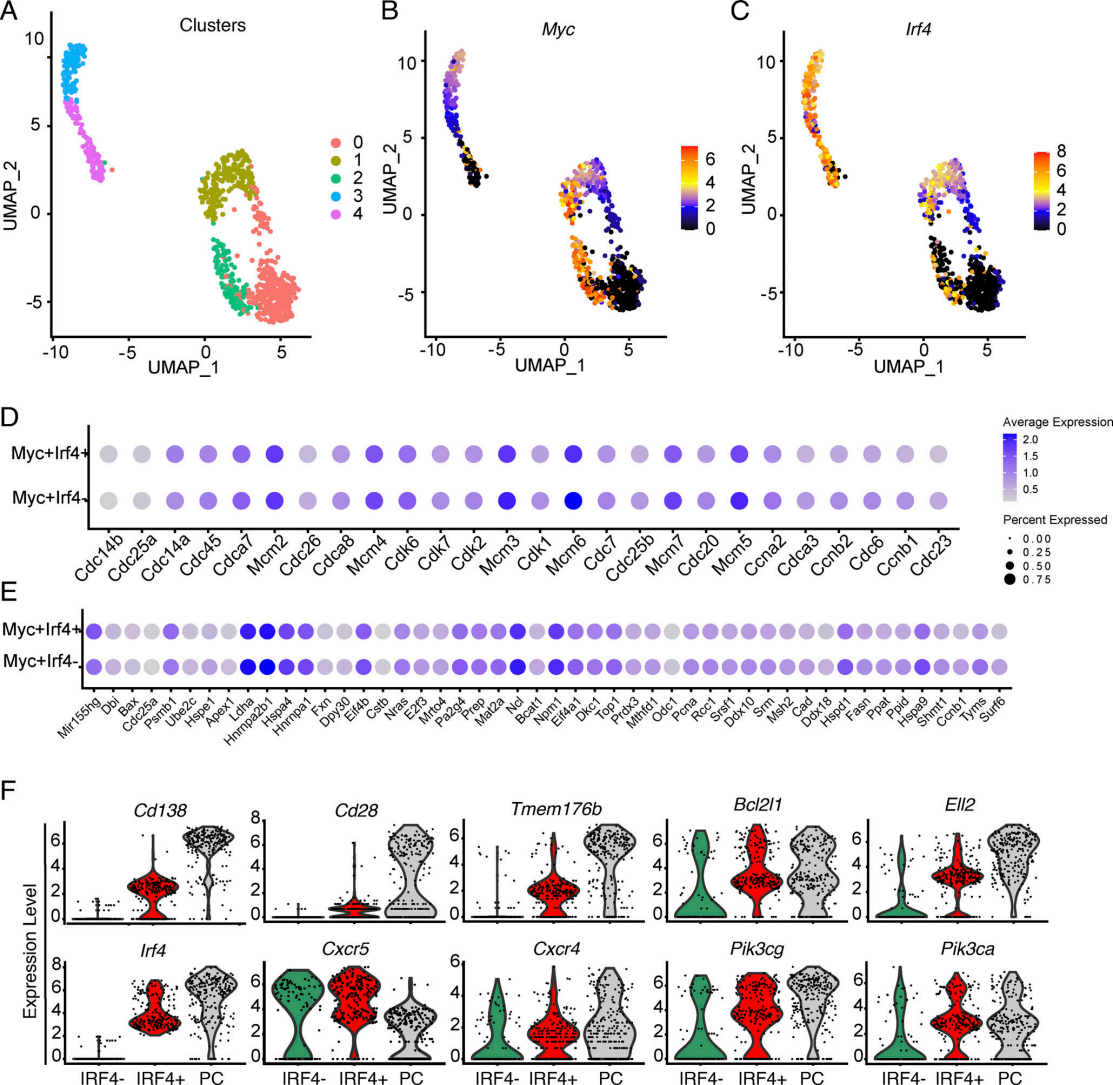
**Gene expression in contemporaneous GC B and PC.** scRNA-seq (SMARTSeq2) performed on sorted LZ, DZ GC B cells, and PCs. **(A)** UMAP visualization outlining the color-coded cluster distribution for LZ, DZ GC B cells, and PCs. **(B and C)** UMAP plots showing relative *Myc* (B) and *Irf4* gene expression (C). **(D and E)** Dot plots show representative cell cycle genes (D) and genes regulated by *Myc* (E) in the indicated cell types (Table S3 and S4). Color intensity shows the expression level and the dot size indicating the percentage of expressing cells in each population. **(F)** Violin plots show expression levels of genes in PC (gray), *Myc*^+^*Irf4*^−^ LZ B cells (green), and *Myc*^+^*Irf4*^+^ pre-PC (red) (see Table S2). Data represent two experiments with four mice each.

**Figure S2.**
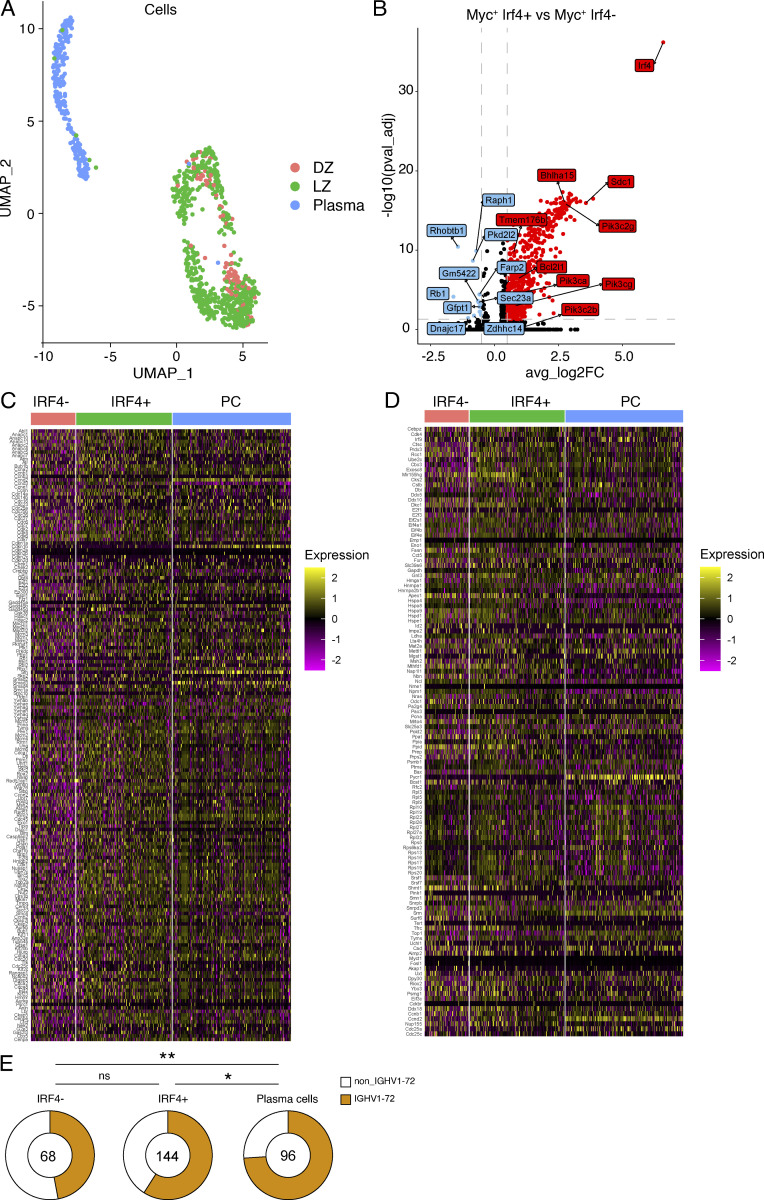
**Transcriptional analysis of***** Irf4*****^+^ and *****Irf4*****^−^ GC B cells.** Related to Fig. 4. **(A)** UMAP visualization outlining the color-coded cluster distribution for LZ, DZ GC B cells, and PCs in the scRNA-seq dataset. **(B)** Volcano plot outlining the differentially expressed genes between LZ Myc-expressing *Irf4*-positive and *Irf4*-negative GC B cells. Genes upregulated by the *Irf4*-positive population are in red whereas genes upregulated by the *Irf4*-negative subset are in blue. **(C and D)** Heatmaps showing the full gene list of cell cycle–related genes upregulated by *Myc* (C) and of genes upregulated by *Myc* (D). **(E)** Pie charts displaying the fraction of cells carrying the IGHV1-72*01 heavy chain gene in each of the three subsets. Statistical significance was calculated using Fisher’s test. * denotes P ≤ 0.05, **P ≤ 0.01, whereas “ns” indicates no significant differences.

The difference in affinity between *S1pr2*-labeled GC B and PC could arise within or outside of GCs. Commitment to the PC fate occurs in the LZ and is associated with *Irf4* expression (Ise et al., 2018; Kräutler et al., 2017; Nutt et al., 2015; Radbruch et al., 2006). To determine whether LZ B cells selected to re-enter the GC or become PC differ with respect to affinity and/or gene expression, we performed high-throughput single-cell RNA sequencing (scRNA-seq) using the Smartseq2 platform which provides a greater sequencing-depth and sensitivity for transcript detection than microfluidics-based technologies such as the 10X Genomics platform (Picelli et al., 2014; Wang et al., 2021a). 14 days after NP-Ova immunization, PCs and LZ B cells were purified from the popliteal LNs by flow cytometry using *Myc*-GFP reporter mice to identify and enrich cells undergoing positive selection (Huang et al., 2008). To uncover distinctive transcriptional characteristics of isolated GC and PC, we visualized single cells in a lower dimensional space according to their gene expression profile using uniform manifold approximation and projection (UMAP) after regressing out cell cycle genes. Clustering analyses assigned the cells to five distinct clusters. Clusters 0, 1, and 2 were primarily composed of LZ cells, and clusters 3 and 4 corresponded to PC (Fig. 4 A and Fig. S2 A). Among LZ cells, c-Myc-expressing cells were distributed in clusters 1 and 2 (Fig. 4, A and B). GC LZ B cells in cluster 1 generally expressed lower levels of *Myc* than the cells in cluster 2, but in addition to *Myc*, cells in cluster 1 also expressed *Irf4* (Fig. 4, A–C). Lower levels of *Myc* found in *Irf4*-expressing cells are consistent with the suppressive effect of *Irf4* on *Myc* expression (Ma et al., 2010; Pathak et al., 2011).

When compared directly, *Myc*^+^*Irf4*^+^ and *Myc*^+^*Irf4*^−^ cells showed numerous transcriptional similarities and differences (Fig. 4, D–F; Fig. S2 B; and Table S2). For example, both cell types expressed genes induced by *Myc*, genes associated with cell division, and *Cxcr4* (Fig. 4, D–F; and Fig. S2, C and D). Out of 115 cell cycle–related genes examined, only four, *Check 2*, *Ccnd2*, *Rb1*, and *E2f2*, were differentially expressed (Fig. 4 D and Table S3). Similarly, out of 51 genes upregulated by *Myc* only three, namely, *Pycr1*, *Cbx3*, and *Ccnd2* were differentially expressed in the two populations (Fig. 4 E and Table S4). However, *Myc*^+^*Irf4*^+^ LZ GC B cells differed from their counterparts in that they also expressed PC–associated genes such as *Sdc1* (CD138), *Cd28*, *Tmem176b*, the UPR-dependent apoptosis suppressor *Bcl2l1* (BCL_XL_; Gaudette et al., 2014), PI3K kinase genes *Pik3ca* and *Pi3kcg* (Shi et al., 2015), as well as *Ell2*, a regulator of splicing required for antibody secretion (Martincic et al., 2009; Fig. 4 F and Fig. S2 B). Thus, *Myc*^+^*Irf4*^*+*^ cells resemble the *Irf4*^hi^ pre-PC subset (Ise et al., 2018; Kräutler et al., 2017) and *Myc*^+^*Irf4*^−^ cells correspond to LZ cells selected to re-enter the DZ (Dominguez-Sola et al., 2012).

**Figure 5.**
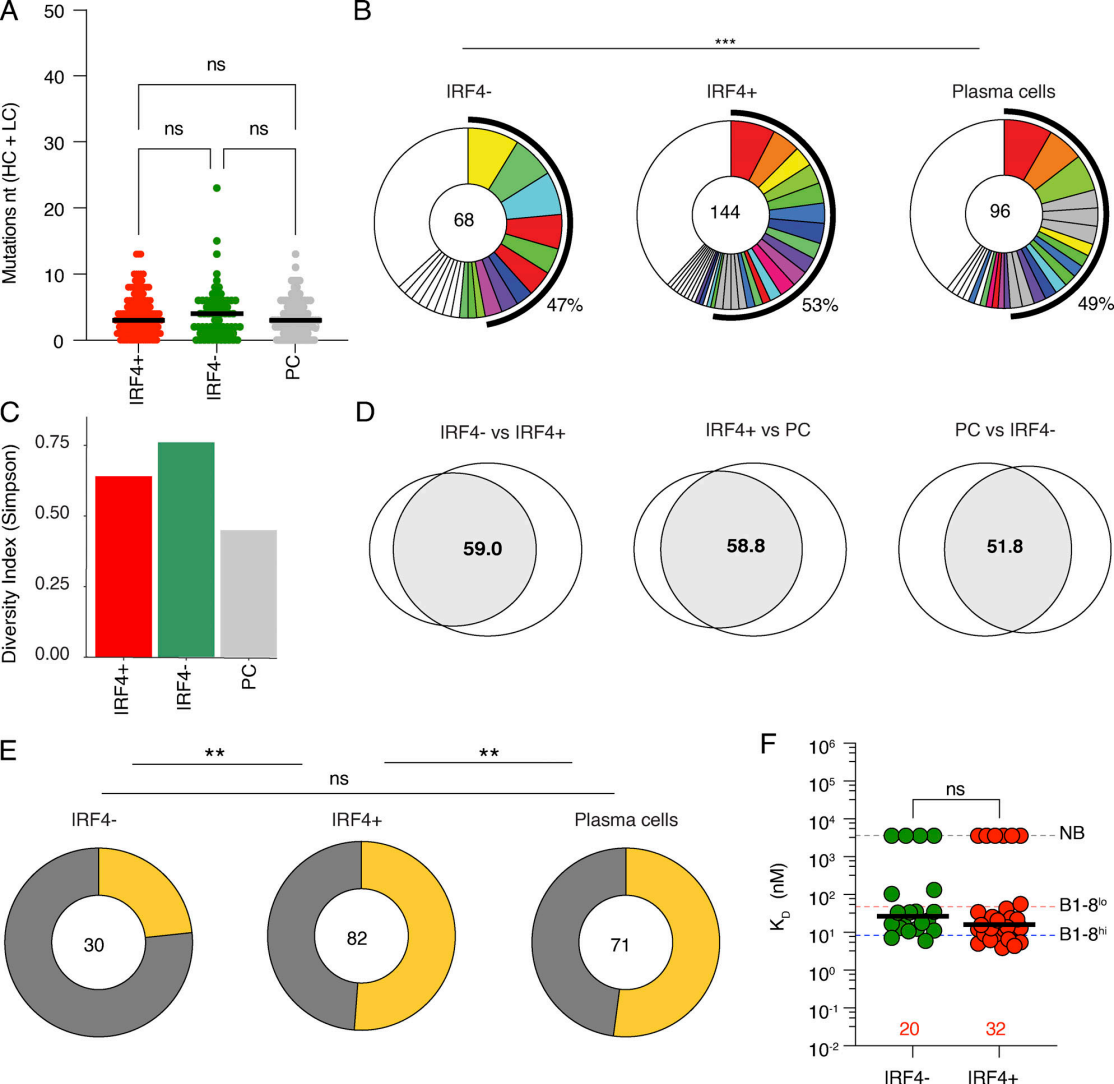
**Antibodies expressed in contemporaneous GC and PC. (A)** Dot plot quantifying the SHM for Ig heavy and light chains (HC + LC) for the cells in Fig. 4 A. Red bar denotes the median value, and “ns” indicates no significant differences. **(B)** Pie charts showing the distribution of antibody sequences in PC, *Myc*-expressing *Irf4*^+^, and *Irf4*^−^ LZ GC B cells. The number inside the circle indicates the number of sequences analyzed. Slice size is proportional to the number of clonally related sequences, with the fraction of clonally expanded sequences summarized as percentage (black outline and associated numbers). Colored slices indicate shared clones between the three populations (same IGHV and IGLV genes, with highly similar complementarity-determining region 3 sequences [CDR3s]), gray slices indicate clones unique to the time point, and white slices indicate sequences isolated only once per time point. Statistical significance was calculated using Fisher’s test. **(C)** Euler plots showing the percentage of clonal overlap between PC, myc-expressing *Irf4*^*+*^, and *Irf4*^−^ LZ GC B cells. The size of the circle is proportional to the total number of sequenced cells per compartment and the size of the overlap region is proportional to the number of shared clones. All sequences including unique sequences and clones with related sequences counted once irrespective of size. The numbers indicate the percentage of clonal overlap. **(D)** Graphs show SDI analysis for all the antibody sequences shown in A and B. **(E)** Pie charts showing the comparison of the fraction of IGHV1-72*01 gene expressing cells that carry any (shown in yellow) or none (shown in gray) of the high-affinity mutations (W33L, K59R, and Y99G) for the three populations. Statistical significance was calculated using Fisher’s test. **(F)** Graph shows monovalent binding affinity K_D_ (nM) determined by BLI for 52 representative Fabs obtained from myc *Irf4*^*+*^ and *Myc Irf4*^*−*^ GC B cells, respectively, on day 14 after immunization. The number of Fabs from each group is written in red. Data represent three experiments with four mice each. A statistical significance of * denotes P ≤ 0.05, **P ≤ 0.01, ***P ≤ 0.001, and ****P ≤ 0.0001, whereas “ns” indicates no significant differences.

To determine how antibodies produced by *Myc*^+^*Irf4*^+^ and *Myc*^+^*Irf4*^−^ LZ B cells might be related to contemporaneous PC present in the same LN, we examined their antibody sequences. The somatic mutation was equivalent in the three populations and they showed similar levels of clonality (Fig. 5, A and B). Consistent with the lineage tracking experiments, but at a level that did not reach statistical significance, diversity was highest in the *Myc*^+^*Irf4*^−^ followed by *Myc*^+^*Irf4*^+^ and PC (Fig. 5 C). In addition, there was also a high degree of clonal overlap between c-Myc^+^IRF4^+^ and c-Myc^+^IRF4^−^ LZ B cells and contemporaneous PC (Fig. 5 D). Moreover, IGHV1-72*01 usage was highest among PC and lowest among *Myc*^+^*Irf4*^−^ LZ cells (Fig. S2 E). Finally, somatic mutations associated with increased affinity (W33L, K59R, Y99G) were similar in *Myc*^+^*Irf4*^+^ and PC and significantly higher than in *Myc*^+^*Irf4*^−^ LZ cells (Fig. 5 E). Thus, the sequence data was consistent with the idea that diversity was highest among c-*Myc*^+^*Irf4*^−^ cells destined to re-enter the DZ and that higher affinity antibody-expressing cells are enriched in the *Myc*^+^*Irf4*^+^ pre-PC and PC compartments (Jacob et al., 1993) (Fig. 5 E and Table S5). To determine the affinities of the antibodies expressed by *Myc*^+^*Irf4*^+^ and *Myc*^*+*^*Irf4*^−^ LZ B cells, we expressed 52 randomly selected Fabs respectively and performed BLI experiments (Fig. 5 F). Fabs from the *Myc*^+^*Irf4*^+^ cells demonstrated a very small relative increase in affinity that did not reach statistical significance compared to their *Irf4*^−^ counterparts (median K_D_ of 16.3 versus 27 nM, respectively).

**Figure 6.**
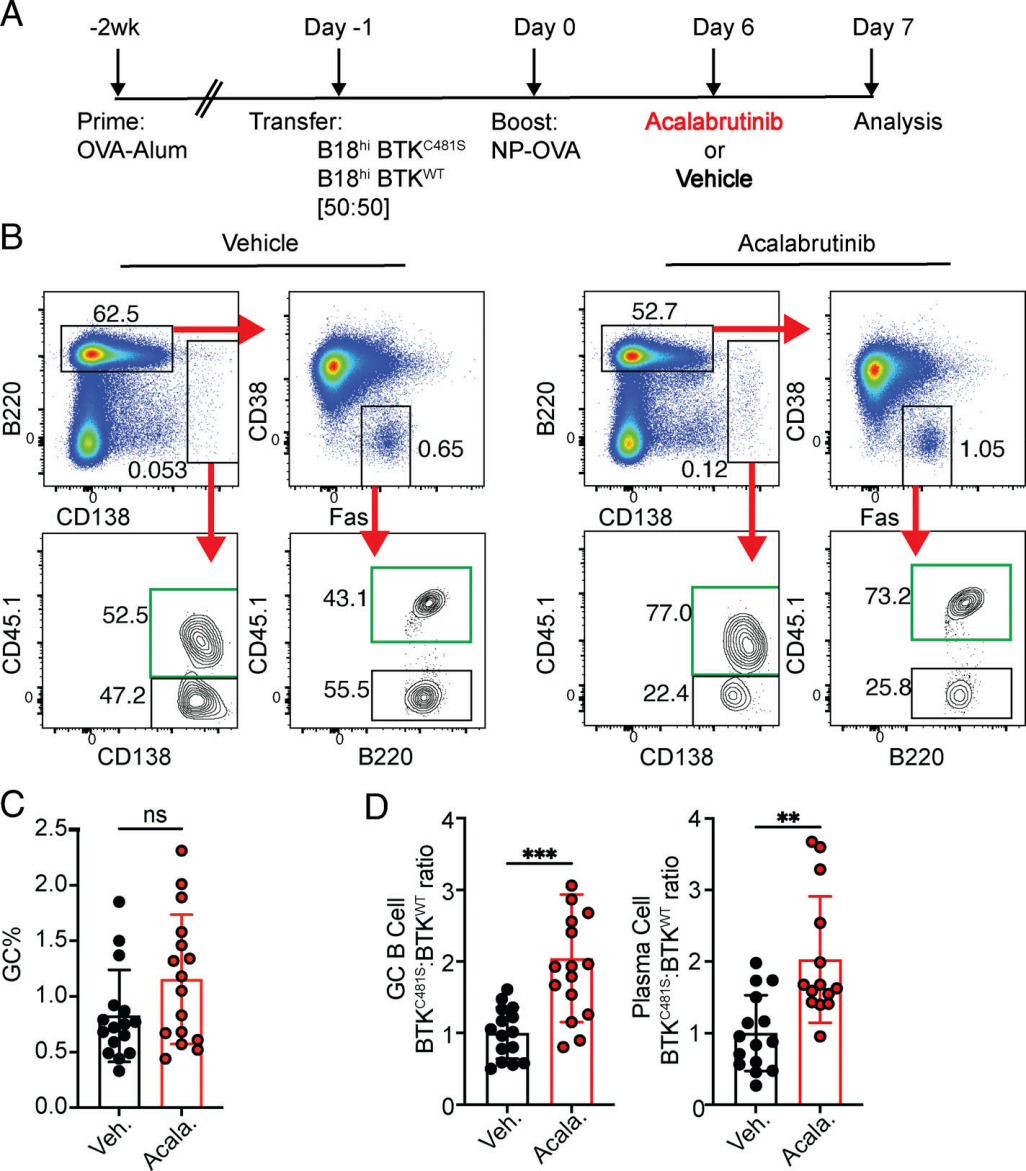
**BCR signaling facilitates PC development. (A)** Experimental setup. CD45.1 B1-8^hi^ BTK^C481S^, B1-8^hi^ BTK^WT^ were transferred into OVA-primed hosts at 50:50 ratio. On day 5 after boosting, 0.03125 mg of acalabrutinib or vehicle was administered by oral gavage, and analysis was performed 24 h later. **(B)** Flow plots show the gating strategy for GC B and PC. Green squares indicate B1-8^hi^ BTK^C481S^ cells (CD45.1^+^). **(C)** Fraction of GC cells in draining LNs in mice treated with vehicle or acalabrutinib. **(D)** Plots show ratios of B1-8^hi^ BTK^C481S^ and B1-8^hi^ BTK^WT^ in GCs (left) and PC (right). In C and D, each dot represents one mouse and ** symbol represents P ≤ 0.01, ***P ≤ 0.001. The results are representative of four independent experiments with three to four mice each.

Positive selection in the GC LZ depends on T follicular helper cells (Victora et al., 2010) and BCR signaling (Chen et al., 2023; Ise et al., 2018; Kräutler et al., 2017; Phan et al., 2006; Victora et al., 2010). Given the similarities between *Myc*^+^*Irf4*^*+*^ and *Myc*^+^*Irf4*^−^ LZ B cells, we asked whether BCR signaling also favors PC development. To do so, we made use of Ig knock-in mice that carry an NP-specific heavy chain and a mutant Bruton’s tyrosine kinase that carries a C481S substitution that makes it resistant to acalabrutinib (B1-8^hi^BTK^C481S^) (Fig. 6 A) (Chen et al., 2023; Woyach et al., 2014). Drug-resistant NP-specific B1-8^hi^BTK^C481S^ and drug-sensitive B1-8^hi^BTK^WT^ B cells were adoptively transferred into OVA-primed mice that were subsequently boosted with NP-Ova (Fig. 6 A). Acalabrutinib was administered at a concentration that inhibited BCR signaling but did not alter GC size or B cell survival (Chen et al., 2023). Consistent with the idea that BCR signaling promotes both c-*Myc*^*+*^*Irf4*^−^ LZ B cell re-entry into the DZ and *Myc*^*+*^*Irf4*^*+*^ pre-PC development, the relative fraction of both drug-resistant GC B and PC increased after acalabrutinib treatment (Fig. 6, B–D) (Chen et al., 2023). The substantial skewing in drug-sensitive cells within the PC compartment likely reflects an effect on pre-PCs.

## Discussion

Current models posit that PC development is restricted by affinity (Phan et al., 2006; Suan et al., 2017b). This idea is based on elegant experiments in which a single amino acid mutation in a transgenic antibody increases its affinity by ∼100-fold (Kräutler et al., 2017; Phan et al., 2006). B cells expressing antibodies that acquired the mutation were preferentially selected into the PC compartment (Kräutler et al., 2017; Phan et al., 2006). We examined PC development in intact mice immunized with NP-OVA and found that although cells expressing higher affinity antibodies are favored to enter the PC compartment, there is significant overlap with cells that are selected to remain in the GC, including cells expressing antibodies with very low affinities that are not measurable under monovalent binding conditions or captured by antigen baiting with fluorescent tetramers (Chen et al., 2023; Viant et al., 2020).

LZ GC B cells are selected to re-enter the DZ to undergo further rounds of division and mutation upon receiving BCR and T follicular helper cell signals that induce *Myc* expression in direct proportion to signal strength (Finkin et al., 2019). Upon DZ re-entry, B cells undergo a fixed number of divisions that are directly proportional to the amount of *Myc* expressed (Finkin et al., 2019; Gitlin et al., 2015; Victora et al., 2010). Over time, several different mechanisms alter GC B cell selection in favor of an increasingly broad range of target epitopes and affinities. These include persistent somatic mutation, continual recruitment of naïve B cells, and antibody masking of initially immunodominant epitopes (Hägglöf et al., 2023; McNamara et al., 2020; Schaefer-Babajew et al., 2023; Schwickert et al., 2007). As a result, all GCs, including those developing in response to simple haptens, evolve over time to include B cells showing high-affinity receptors and a diverse collection of cells showing lower levels of affinity that contribute to a diverse B cell memory compartment (Inoue and Kurosaki, 2023; Shinnakasu et al., 2016; Viant et al., 2020).

PC precursors share many of the transcriptional programs found in LZ GC B cells selected to re-enter the DZ; however, the two cell types differ in some important respects. Like LZ cells re-entering the DZ, PC precursors express *Myc* and the transcriptional program induced by c-Myc including cell division genes and *Cxcr4*. Consistent with the shared transcriptional program, our experiments demonstrate that both GC B and PC selection are dependent upon BCR signaling. The similarities suggest a mechanistic explanation for the clonal overlap between the GC and PC compartment and support the idea that the final steps in PC commitment occur in the DZ (Kräutler et al., 2017; Radbruch et al., 2006; Radtke and Bannard, 2019).

Despite these similarities, the GC and the pre-PC compartments differ in their transcriptional profiles and their antibody repertoires. The transcriptional differences between the two compartments are in part related to *Irf4 *expression in pre-plasma cells resulting in the expression of many of the genes required for plasma cell differentiation (Ise et al., 2018; Nutt et al., 2015; Radbruch et al., 2006). Antibody repertoires expressed by GC B and plasma cells differ in that the antibodies expressed by plasma cells are less diverse and show higher affinity. Our experiments indicate that affinity-based selection is initially manifested in the GC and that BCR affinity and signaling contribute to this cell fate decision in the LZ of the GC. The difference in affinity between cells destined to remain in the GC and pre-plasma cells are small, but over time, GCs produce a relatively diverse plasma cell compartment that is selected for higher affinity in a manner consistent with the initial description of increasing serum antibody affinity after immunization (Eisen and Siskind, 1964). Given the relatively small initial differences between LZ GC B cells selected for DZ re-entry and plasma cell development, it is likely that additional yet-to-be-determined factors contribute to high-affinity plasma cell accumulation.

In conclusion, modest differences in affinity distinguish LZ B cells selected to re-enter the DZ from pre-PC. These differences accumulate over time accounting for the increases in serum antibody affinity with time after immunization (Eisen and Siskind, 1964). Similarities between the GC and PC compartments can be accounted for by a shared transcriptional program that is anchored by c-Myc. Thus, although the antibodies expressed by PC are somewhat restricted compared with those found in GCs, there is significant overlap between the two compartments which favors the production of the type of diversified immune response that is required to deal with evolving pathogens.

## Materials and methods

### Mice

S1pr2-CreERT2 mice were generously provided by T. Kurosaki (Osaka University, Osaka, Japan) (Shinnakasu et al., 2016). Rosa-tdtomato (Ai14; Rosa-CAG-LSL-tdTomato-WPRE stock no. 007914) mice were purchased from the Jackson Laboratory. Mice used in this study ranged from 8 to 12 wk old. Wild-type C57BL/6J mice were purchased from the Jackson Laboratory. *Myc*-GFP mice were as described (Dominguez-Sola et al., 2012). All animal procedures were performed in accordance with the protocols approved by The Rockefeller University institutional animal care and use committee.

### Immunization and tamoxifen treatment

Footpad immunizations were performed with 25 μl of PBS containing 12.5 μg of NP-OVA (Biosearch Technologies, N-5051-100) precipitated in aluminum hydroxide gel adjuvant (Alhydrogel) (Invivogen, vac-alu-250) at a 2:1 ratio. Activation of the Cre recombinase in the S1pr2-ERT2cre mice was induced by a single oral administration (gavage) of 12 mg tamoxifen (Sigma-Aldrich, T5648) in 200 μl of corn oil (Sigma-Aldrich, C8267) at the indicated time point.

### Flow cytometry

Popliteal LNs were homogenized using disposable micropestles and collected in FACS buffer (PBS 1×, 2% FBS, 2 mM EDTA) on ice. Single-cell suspensions were obtained by mechanical disruption through a 70-mm cell strainer (BD Biosciences). Erythrocytes were lysed with 1 ml of ammonium–chloride–potassium lysing buffer (GIBCO). After incubation with 5 μg/ml of anti-CD16/32 (rat mAb 2.4G2; mouse Fc block; BD Biosciences, 553142) for 15 min at 4°C, cell surface antigens were stained for 30 min at 4°C. When a biotin antibody was used, the cells were then incubated with a streptavidin-fluorophore conjugate for 20 min at 4°C. Flow cytometric analysis was performed on a BD FACS Symphony.

Antibodies: CD138 (BioLegend, clone 281-2, cat. 142523), TACI (Thermo Fisher Scientific, clone ebio8F10-3 on either APC or BioLegend; clone 8F10 on PE), CD95/Fas (BD Biosciences, clone JO2, 556653), B220 BUV805 (BD Biosciences, clone RA3-6B2, 748867), CD38 BV605 (BD Biosciences, clone 90, 740361). To exclude non-B cells or non-PCs, anti-CD4-eF780 (Thermo Fisher Scientific, RM4-5, 47-0042-82), anti-CD8-eF780 (Thermo Fisher Scientific, clone 53-6.7, 47-0081-82), Anti-NK1.1-eF780 (Thermo Fisher Scientific, clone PK136, 47-5941-82), Anti-F4/80-eF780 (Thermo Fisher Scientific, clone BM8, 47-4801-82), and anti-Ly6G (Thermo Fisher Scientific, clone 1A8-Ly6g, 47-9668-82). Dead cells were identified and excluded using live/dead Zombie NIR (BioLegend, 423106). For sorting DZ and LZ GC B cells sorting (Fig. 4), anti-Igλ1 (BD Biosciences, clone R26-46, 744526), CXCR4-PE (BD Biosciences, clone 2B11, 551966), and CD86-BV711 (BD Bioscience, clone GL1, 740688) were used. For enrichment of positively selected GC B cells, c-Myc-GFP LZ GC B cells were FACS-sorted in addition to the GFP-negative GC B cells.

### Single-cell BCR sequencing

This procedure was performed as previously described in STAR protocols (Viant et al., 2021a). GC B cells and PCs were sorted using a BD FACS Symphony S6 into 96-well plates containing 5 μl lysis buffer (turbocapture lysis [TCL] buffer [Qiagen, 1031576] 1% 2-β-mercaptoethanol [Sigma-Aldrich, M3148]) and immediately frozen at −80°C. Single-cell RNA was purified using magnetic beads (RNAClean XP, Beckman Coulter, A63987) following the manufacturer’s instructions. RNA was eluted from the magnetic beads with 11 μl of a solution containing random primers (14.5 ng/μl, Invitrogen, 48190-011), tergitol (0.5% of NP-40 70% in H_2_O, Sigma-Aldrich, NP40S), and RNase inhibitor (0.6 U/μl, Promega, N2615) in nuclease-free water (Qiagen) and then incubated at 65°C for 3 min. cDNA was subsequently synthesized by reverse transcription (RT) with 7 μl of a solution containing: SuperScript III Reverse Transcriptase, 5× buffer, dithiothreitol (SuperScript III Reverse Transcriptase, Invitrogen, 18080-044, 10,000 U), deoxynucleotide triphosphate (25 mM) RNase inhibitor (0.6 U/μl, Promega, N2615) in nuclease-free water (Qiagen). Thermal cycler incubations for RT reaction are at 1× (42°C, 10 min; 25°C, 10 min; 50°C, 60 min; 94°C, 5 min). cDNA was stored at −20°C or immediately used for antibody gene amplification by nested polymerase chain reaction (PCR) after the addition of 10 μl of nuclease-free water. After purification, heavy chain and light chain PCR products were Sanger sequenced and subsequently analyzed using MacVector and Geneious Prime (v.2022.1.1), as well as the bioinformatics pipeline detailed below in the “Fab production” section.

### Fab production

The PCR products of antibody heavy chain and light chain genes were purified and Sanger-sequenced (Genewiz). Subsequently, *ab1 files were analyzed using the IgPipeline previously described (https://github.com/stratust/igpipeline/tree/igpipeline2_timepoint_v2) (Cho et al., 2021; Gaebler et al., 2021; Wang et al., 2021b).

V(D)J sequences were ordered as eBlocks (IDT) containing short homologies at both ends for Gibson assembly and cloned into human Fab IgG1 or human IgK or human IgL2 expression vectors using the NEB Hifi DNA Assembly mix (NEB, Cat#E2621L). Plasmid sequences were verified by Sanger sequencing (Genewiz). His6-tagged Fabs, and κ and λ light chains were expressed by transient transfection in Expi293F cells (Thermo Fisher Scientific) and purified using Ni Sepharose 6 Fast flow resin (Cytiva).

### ELISA

ELISA assays to measure Fab binding to NP-Ova were performed by coating high-binding 96-half-well plates (Corning, 3690) with 50 μl per well of a 1 μg ml^−1^ protein solution in PBS overnight at 4°C. The plates were washed six times with washing buffer (1× PBS with 0.05% Tween-20, Sigma-Aldrich) and incubated with 170 μl of blocking buffer per well (1× PBS with 2% BSA and 0.05% Tween-20, Sigma-Aldrich) for 1 h at room temperature. Immediately after blocking, the tested Fabs were added to PBS and incubated for 1 h at room temperature. Fab was tested at a starting concentration of 10 μg ml^−1^ and 11 additional threefold serial dilutions. The plates were washed six times with washing buffer and then incubated with anti-human Fab IgG secondary antibody conjugated to horseradish peroxidase (HRP) (Jackson Immuno Research, 109-036-088) in blocking buffer at a 1:5,000 dilution. The plates were developed by the addition of the HRP substrate 3,3′,5,5′-tetramethylbenzidine (Thermo Fisher Scientific) for 3 min. The developing reaction was stopped by adding 50 μl of 1 M H_2_SO_4_ and absorbance was immediately measured at 450 nm using an ELISA microplate reader (FluoStar Omega, BMG Labtech) with Omega and Omega MARS software for analysis. B1-8^lo^ Fab was used as a normalizer control sample and its dilutions were included on each plate. EC_50_ were calculated using a four-parameter nonlinear regression model (GraphPad Prism v.9.3) with the following settings: [agonist] vs. response -- variable slope (four parameters); bottom = 0; Hillslope > 0; top = experiment-specific upper plateau of the normalizer control antibody or plasma sample reaching saturation for at least three consecutive dilution steps. The curve fit was constrained to an upper limit that corresponds to the maximal optical density achieved by the known normalizer control to limit interplate/interexperiment variability (batch effects). All reported EC_50_ values are the average of at least two independent experiments.

### BLI

BLI measurements were performed using a ForteBio Octet Red96 (Sartorius) as previously described on 96-well assay plates (Greiner Bio-One, 655209) (Chen et al., 2023). The plates were assayed at 30°C with shaking at 1,000 rpm. Monovalent binding assays were performed using high precision Streptavidin Biosensors (Sartorius) loaded with NIP_16_Ova-biotin (Biosearch Technologies, 200 nM). Curve fitting was performed using a fast 1:1 binding model and the data analysis software from ForteBio. Mean K_D_ were determined by averaging all binding curves that matched the theoretical fit with an *R*^2^ value of ≥0.8. For avidity measurements of Fabs that did not show binding by monovalent BLI assays, anti-human FAB-CH1 biosensors (FAB2G, Sartorius) were loaded with (monoclonal) Fabs fragments (100 nM) diluted in 1× kinetics buffer and assayed with NP_16_Ova at 5 and 1 µg ml^−1^. B1-8^hi^ and B1-8^lo^ were used as positive control Fab (for binding) and HuCal Fab-MH (Bio-Rad, HCA051) was used as negative control.

### Cell-sorting strategies

Cell sorting for single-cell RBCR sequencing and SMARTSeq2 was performed on a BD FACS Symphony Sorter as described in the “Flow cytometry” section. After gating on live singlets, PC were defined as CD138^+^ TACI^+^ and negative for lineage (Lin) negative markers. The lineage markers used to exclude cells included F4/80, CD4, CD8, Ly6G, and NK1.1. GC B cells were defined as lin^−ve^ B220^+^ CD38^−ve^ Fas^+^ cells. This was followed by gating based on S1pr2-labeling via ZsG reporter or TdT reporter (Shinnakasu et al., 2016). For gating on LZ GC B cells, after gating on GC B cells, CD86^hi^ CXCR4^lo^ cells were sorted and for DZ GC B cells, CXCR4^hi^ CD86^lo^ exactly as described (Chen et al., 2023). In the SMARTSeq2 sequencing experiments in Fig. 4, enrichment of c-Myc^+^ cells was achieved using the c-Myc-GFP reporter by gating on LZ GFP^+^ GC B cells.

### scRNA-seq (SMARTSeq2)

Single GC B cells and PCs from two independent experiments were sorted into 96-well plates containing 5 μl TCL buffer (Qiagen), supplemented with 1% β-mercaptoethanol (Sigma-Aldrich) using a BD FACS Symphony S6 sorter. Single-cell RNA was purified using magnetic beads (RNAclean XP, Beckman Coulter). RNA was reverse transcribed to cDNA using oligodT primers and Maxima H− reverse transcriptase (Thermo Fisher Scientific) to generate “template-switched” cDNA and amplified as previously described (Islam et al., 2014; Picelli et al., 2014; Trombetta et al., 2014). Libraries were prepared using an Illumina DNA Prep kit (Illumina), indexed using IDT for Illumina Index Sets (Illumina), and sequenced on an Illumina NovaSeq platform (Rockefeller University Genomics Resource Center).

### Smartseq2 computational analysis

We employed STAR (Dobin et al., 2013) to map and quantify the sequence reads aligned to the *Mus musculus* reference genome GRCm38. For scRNA-seq analyses, cluster identification, and differential gene expression analyses, we used Seurat (v.4.3.0) (Stuart et al., 2019). BCR sequences were reconstructed using TRUST4 (Song et al., 2021). Heavy and light chains derived from the same cell were subsequently paired and clonotypes were assigned using the publicly available pipeline at GitHub (https://github.com/stratust/igpipeline/tree/igpipeline2_timepoint_v2). Gene sets consisted of 115 cell cycle–related genes and 51 genes upregulated by c-Myc (Tables S2 and S3; https://www.gsea-msigdb.org/gsea/msigdb/human/geneset/DANG_MYC_TARGETS_UP.html). Gene expression and statistical information were calculated from the differential expression analyses by Seurat. R programming language was used to apply Fisher’s exact test to evaluate whether there was a statistically significant change in the immunoglobulin clonal distribution and distribution of affinity-enhancing mutations between c-Myc^+^IRF4^+^ and c-Myc^+^IRF4^−^ LZ B cells.

### Online supplemental material

Fig. S1 (related to Fig. 1) shows that popliteal LNs are devoid of PC in the absence of immunization compared to immunized mice on day 14. Fig. S2 displays genes upregulated by Irf4-positive GC B cells compared with the Irf4-negative subsets and similar genes (cell cycle–related and Myc-upregulated). It also quantifies the fraction of cells carrying IGHV1-72*01 in the subsets compared. Table S1 shows BCR sequences for each expressed Fab, ELISA, and K_D_ results related to Fig. 3. Table S2 shows transcriptional profiles of Myc-expressing Irf4-positive and Irf4-negative LZ GC B cells. Table S3 shows the list of cell cycle–related genes. Table S4 shows the list of genes upregulated by Myc in Ir4-positive, Irf4-negative GC B cells, and PCs (Tables S2, S3, and S4 are related to Fig. 4). Table S5 details BCR sequences for LZ Myc-expressing IRF4-positive, IRF4-negative, and PCs (related to Fig. 5)

## Supplementary Material

Table S1 lists the heavy and light chain genes for each expressed Fab, ELISA EC50 results, and K_D_ values from monomeric (affinity) BLI analysis as well as the high- and low-affinity mutations present in Fabs carrying IGHV1-72*01 gene.

Table S2 lists the differentially expressed genes between *Myc*-expressing *Irf4*-positive LZ GC B cells and *Irf4*-negative LZ GC B cells.

Table S3 lists the cell cycle–related genes.

Table S4 lists the genes upregulated by *Myc* in *Irf4*-positive LZ GC B cells, *Irf4*-negative LZ GC B cells, and PCs.

Table S5 lists the antibody sequences for LZ Myc-expressing IRF4-positive, IRF4-negative, and plasma cells.

## Data Availability

Ig sequences for ELISA and EC_50_ (in Fig. 3) are provided in Table S1. Ig sequences discussed in analyses in Fig. 5 and Fig. S2 are provided in Table S5. The differentially expressed genes between *Myc*-expressing *Irf4*-positive LZ GC B cells and *Irf4*-negative LZ GC B cells are listed in Table S2. Gene expression profiles for c-Myc–regulated genes and cell cycle genes (Fig. 4) are provided in Table S3 and Table S4, respectively. The raw sequencing data and computer scripts associated with Fig. 1 and Fig. 5 have been deposited at GitHub (https://github.com/stratust/igpipeline/tree/igpipeline2_timepoint_v2). The computer code to process the antibody sequences is available on GitHub (https://github.com/stratust/igpipeline/tree/igpipeline2_timepoint_v2). scRNA-seq (Smartseq2) data from this study (Fig. 4 and Fig. 5) are available at the Gene Expression Omnibus under accession no. GSE246382.
