# Correction: Role of affinity in plasma cell development in the germinal center light zone

**DOI:** 10.1084/jem.2023183801082024c

**Published:** 2024-01-16

**Authors:** Mohamed A. ElTanbouly, Victor Ramos, Andrew J. MacLean, Spencer T. Chen, Maximilian Loewe, Sandra Steinbach, Tarek Ben Tanfous, Brianna Johnson, Melissa Cipolla, Anna Gazumyan, Thiago Y. Oliveira, Michel C. Nussenzweig

Vol. 221, No. 1 | https://doi.org/10.1084/jem.20231838 | November 8, 2023

The authors regret that the Fig. 3 legend was incorrect in their originally published article. The original panel A description has been deleted, and the panel letters have been relabeled accordingly. The figure and corrected legend appear here, with changes in italics and underlined. This correction does not alter the original conclusions of the article. Figure citations and the figure itself remain unchanged. The errors appear in print and in PDFs downloaded before January 8, 2024.

**Figure 3.**
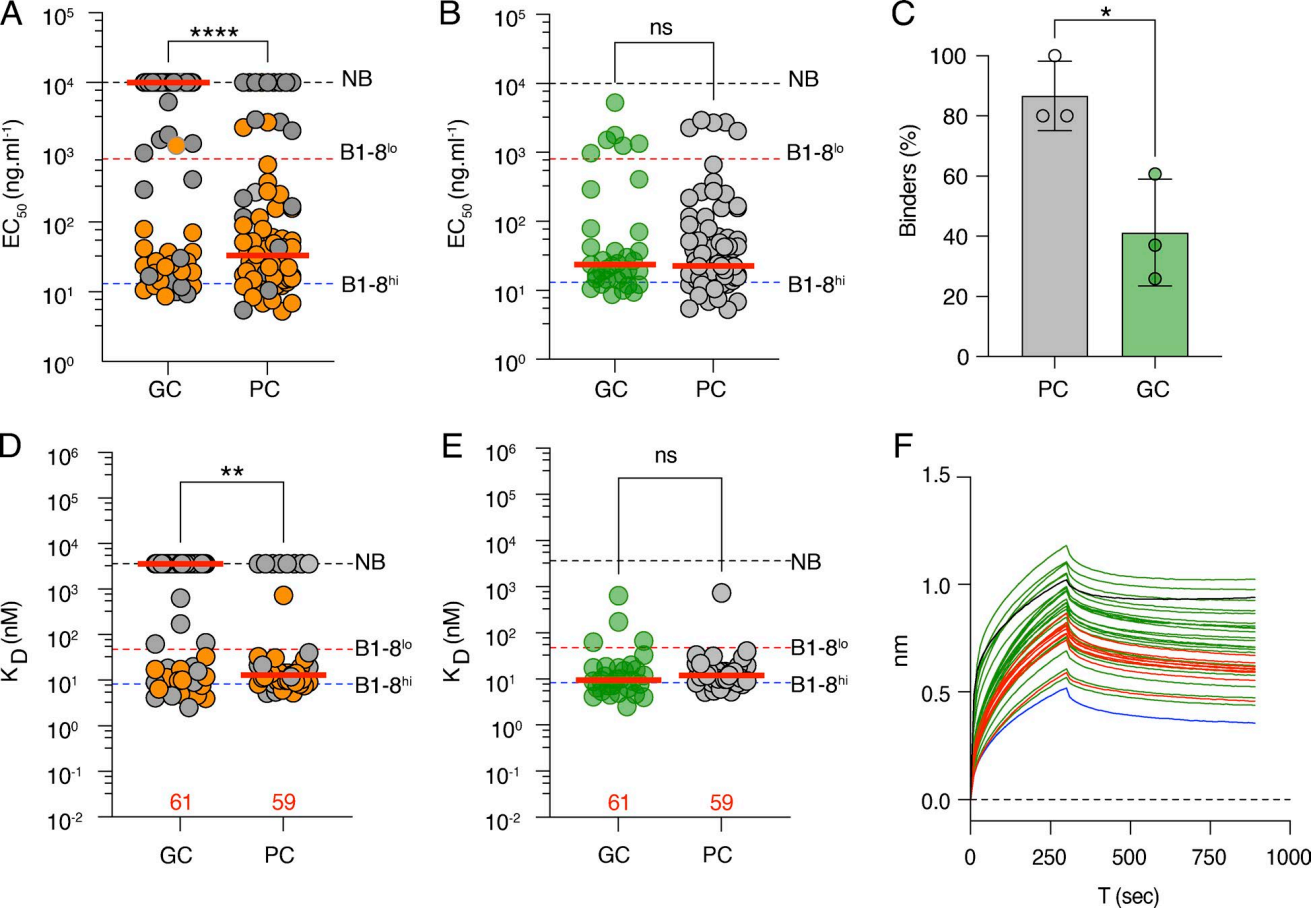
**Antibody affinity.** ***(A)*** Dot plot showing ELISA EC_50_ binding for 81 and 86 representative Fabs expressed and tested from GC B and PC, respectively, on day 21 after immunization. VH1-72*01 antibodies are in orange, and all other antibodies are in gray. ***(B)*** As in *A*, but excluding antibodies that do not bind. GC B (green); PC (gray). ***(C)*** Bar graph shows the fraction of all Fabs tested that show demonstrable binding in ELISA. Statistical significance was determined using an unpaired *t* test. ***(D)*** Graph shows monovalent binding affinity K_D_ (nM) determined by BLI for 61 and 59 representative Fabs obtained from GC B and PC, respectively, on day 21 after immunization. Color scheme as in *A*. ***(E)*** As in *D*, but excluding Fabs that do not bind. The red horizontal bars in *A–E* represent the median values. Statistical significance was determined using two-tailed Mann–Whitney U-tests comparing the differences between GC and PC EC_50_ in *A* and *B*, and K_D_ in *D* and *E*. ***(F)*** Graphs show BLI traces for 43 GC B (green) and PC (red) Fabs that show no demonstrable binding activity under monovalent conditions (*D*) tested under multivalent conditions. B1-8lo (black) and negative control Fab (blue). All experiments in this figure were performed at least in duplicate. The red bar denotes the median value and the statistical significance of * denotes P ≤ 0.05, **P ≤ 0.01, ***P ≤ 0.001, and ****P ≤ 0.0001, whereas “ns” indicates no significant differences.

